# Expression and purification of tau protein and its frontotemporal dementia variants using a cleavable histidine tag

**DOI:** 10.1016/j.pep.2016.09.009

**Published:** 2017-02

**Authors:** Thomas K. Karikari, Alexandra Turner, Robert Stass, Leonie C.Y. Lee, Bethany Wilson, David A. Nagel, Eric J. Hill, Kevin G. Moffat

**Affiliations:** a School of Life Sciences, University of Warwick, Coventry CV4 7AL, UK; b Midlands Integrative Biosciences Training Partnership, University of Warwick, Coventry CV4 7AL, UK; c Aston Research Center for Healthy Ageing, School of Life and Health Sciences, Aston University, Birmingham B4 7ET, UK

**Keywords:** Tau, Tauopathies, Microtubule-associated protein tau, Alzheimer's disease, FTPD-17, Expression, Purification

## Abstract

Recombinant tau protein is widely used to study the biochemical, cellular and pathological aspects of tauopathies, including Alzheimer's disease and frontotemporal dementia with Parkinsonism linked to chromosome 17 (FTPD-17). Pure tau in high yield is a requirement for *in vitro* evaluation of the protein's physiological and toxic functions. However, the preparation of recombinant tau is complicated by the protein's propensity to aggregate and form truncation products, necessitating the use of multiple, time-consuming purification methods. In this study, we investigated parameters that influence the expression of wild type and FTPD-17 pathogenic tau, in an attempt to identify ways to maximise expression yield. Here, we report on the influence of the choice of host strain, induction temperature, duration of induction, and media supplementation with glucose on tau expression in *Escherichia coli*. We also describe a straightforward process to purify the expressed tau proteins using immobilised metal affinity chromatography, with favourable yields over previous reports. An advantage of the described method is that it enables high yield production of functional oligomeric and monomeric tau, both of which can be used to study the biochemical, physiological and toxic properties of the protein.

## Highlights

• Factors influencing the expression of wild type and FTPD-17 pathogenic tau were investigated in an attempt to maximise yield.
• Soluble monomeric tau expression level was highest at 37 °C compared to 20 °C and 25 °C.
• Media supplementation with 0.2% glucose did not significantly influence monomeric tau expression levels.
• Circular dichroism confirmed that the purified tau proteins were mostly unfolded, with negative peaks around 200 nm.
• The circular dichroism peaks shifted towards 220 nm following the preparation of Alzheimer-like filaments, suggesting secondary structure re-orientation towards β-sheets.
• The tau proteins adopted classical fibrillisation similar to filaments isolated from the brains of Alzheimer's disease patients.

## 1 Introduction

Tauopathies are a major neurodegenerative health concern worldwide. These include Alzheimer's disease, frontotemporal dementia with Parkinsonism linked to chromosome 17 (FTPD-17), Pick's disease, progressive supranuclear palsy and corticobasal degeneration [1]. A common feature shared by these diseases is the abundance of tau-positive neurofibrillary tangles (NFTs) that define neuropathology [1], [2]. While tau is involved in microtubule (MT) assembly and stabilisation in physiological conditions, dysfunctional tau has been implicated in tauopathy-associated NFTs resulting from the accumulation of insoluble toxic aggregates [3].

Recombinant tau closely mimics important properties of the naturally-occurring human protein. To this end, recombinant tau has been used extensively to study the molecular, biochemical and cellular aspects of the protein's functions, including (i) binding to MTs in physiological and pathological conditions (ii) tau-tau interactions leading to their aggregation into toxic paired helical filaments (PHFs) that are similar to those isolated from the brains of Alzheimer's disease patients, and (iii) the transcellular spread of this toxicity in affected organisms [4], [5], [6], [7], [8], [9], [10]. The binding of tau to MTs is disrupted in disease and also by the presence of specific FTPD-17 mutations some of which can enhance the protein's aggregation into PHFs [6], [9], [11], [12]. Furthermore, truncated tau constructs such as the repeat domains are *aggregation*-*prone* and demonstrate enhanced aggregation kinetics compared to the full-length isoforms [13], [14]. The tau four-repeat domain (K18 construct), for example, is sufficient for the protein's polymerisation into PHFs [4]. When exogenously supplied, this tau fragment can be internalised by cells, engaging endogenous proteins to form toxic aggregates which spread in prion-like cell-to-cell transmission [7], [8], [15], [16].

High-yield expression of pure tau is critical for *in vitro* tauopathy studies. However, the protein's expression and purification are complicated by its high aggregation and truncation tendencies, and time-consuming and expensive methodologies [17]. Here, we sought to identify parameters that would ensure optimal expression of tau K18 and its FTPD-17 variants V337M and N279K. The following expression conditions were investigated: the choice of *E*. *coli* host strain, induction temperature, duration of induction, and the presence of glucose in the culture medium. Subsequently, the newly established conditions were utilised for the large-scale protein expression and purification. The purified proteins were characterised through circular dichroism analysis of secondary structures and the assembly of Alzheimer-like PHFs.

## 2 Materials and methods

### 2.1 Cloning of K18 into pProEx plasmid

**Fig. 1:**
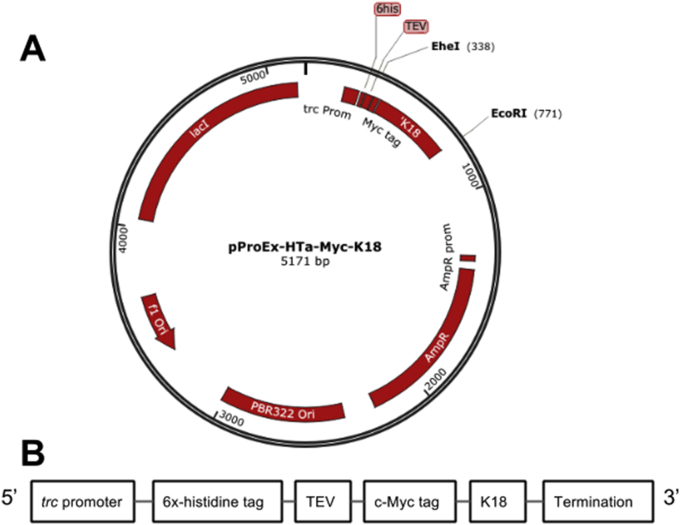
**The wild type K18 expression plasmid used in this study. (A)** A schematic representation of the pProEx-HTa plasmid into which the K18 tau construct was cloned. The *Ehe*I and *Eco*RI restriction sites between which K18 was cloned are shown, as well as the genes encoding the LacI repressor (*lacI*), ampicillin resistance (AmpR), and origins of replication (f1Ori and pBR322 Ori). **(B)** Essential elements of the pProEx-HTa-Myc-K18 plasmid are indicated. These include a *trc* promoter, a poly-histidine tag, a TEV cleavage site and a c-Myc tag followed by K18.

A pProEx-HTa-Myc-K18 plasmid was constructed for the recombinant expression of K18 tau and two FTPD-17 variants. In each case, the K18 sequence was preceded by the Tobacco Etch Virus (TEV) protease-recognition sequence ENLYFQG fused at the N-terminus to a poly-histidine tag and a c-Myc tag (Fig. 1). This arrangement enabled large-scale protein purification using IMAC and subsequent TEV protease cleavage.

The coding sequence of K18 was generated by polymerase chain reaction using pCMV-FLAG-tau as template (a gift from Dr. Calum Sutherland, University of Dundee, UK) and cloned between the *Ehe*I and *Eco*RI sites of the pProEx-HTa plasmid (Invitrogen) containing ampicillin-resistance and *lacI-*encoding genes (Fig. 1). Expression was controlled by a *trc* promoter, and initiated with the addition of isopropyl β-D-1 thiogalactopyranoside (IPTG). Plasmid sequences were confirmed by DNA sequencing (GATC Biotech AG, Konstanz, Germany).

### 2.2 Site-directed mutagenesis (SDM)

The Q5^®^ SDM kit (New England Biolabs) was utilised to create the N279K and V337M pathogenic mutations in the K18 wild type (WT) tau, according to manufacturer's instructions, using primers listed in Table 1 (designed using the NEBaseChanger™ programme http://nebasechanger.neb.com, version 1.2.3). Plasmids were transformed into competent NEB-5α *E*. *coli* cells (a DH5α derivative) and grown overnight in Luria-Bertani (LB) broth containing 100 μg/ml ampicillin in a shaking incubator with agitation (180 rpm) at 37 °C. Plasmid DNA was isolated using the QIAprep Spin Miniprep kit (QIAGEN GmbH, Hilden, Germany) according to manufacturer's instructions. The presence of the mutations was confirmed by DNA sequencing as described above, using the primers listed in Table 2, following which plasmid DNA was transformed into BL21(DE3)*pRosetta cells.

### 2.3 Transformation of competent cells

Five nanograms of plasmid DNA was added to 50 μl competent BL21(DE3)*pRosetta cells and incubated on ice for 30 min. Cells were heat-shocked at 42 °C for 30 s, followed by 2 min incubation on ice. 950 μl LB broth was added and the mixture incubated at 37 °C for 1 h. 40 μl of the mixture was plated on LB agar supplemented with ampicillin (100 μg/ml) and chloramphenicol (35 μg/ml) and incubated overnight at 37 °C.

### 2.4 Expression of the K18 tau constructs

Single colonies were inoculated into 10 ml selective LB broth and grown overnight at 37 °C. The following morning, 1 ml of the overnight culture was inoculated into 50 ml selective LB broth and grown at 37 °C with 180 rpm agitation. Cultures were grown until reaching OD_600_ = 0.6–0.7, induced with 0.5 mM IPTG and grown for a further 3 h. 1.5 ml aliquots were centrifuged at ∼11,000×*g* for 1 min, the pellet resuspended in 100 μl phosphate buffered saline (PBS) and sonicated for 5 s at 10% power and re-centrifuged at ∼11,000×*g* for 5 min. All sonication was done in a Bandelin Sonopuls 2070 sonicator. The soluble (supernatant) fraction was pipetted into an Eppendorf tube for gel electrophoresis. The pellet was resuspended in 500 μl PBS, sonicated (10% power, 5 s) and centrifuged (∼11,000×*g* for 5 min). The resultant pellet was resuspended in 150 μl PBS, producing the insoluble fraction. Tau expression in the soluble and insoluble fractions was analysed by sodium dodecyl sulphate polyacrylamide gel electrophoresis (SDS-PAGE) and Western blotting (WB) as described below.

### 2.5 SDS-PAGE and WB

Protein samples were separated on standard 15% Tris-glycine SDS-PAGE gels with (reducing gels) or without (non-reducing gels) β-mercaptoethanol and 5 min heating at 95 °C. Samples were analysed against a protein ladder (P7712 or P7712S from New England BioLabs, size range = 11–245 kDa) for 35 min at 200 V in a Biorad Mini-PROTEAN Tetra system (BioRad Laboratories, California, USA) and stained with Instant Blue (Coomassie-based stain from Expedeon, Cambridge, UK) for 1 h at room temperature (RT) with no washing steps required and imaged using a SynGene G-Box imaging system. To identify tau-positive bands, WB was performed prior to antibody detection as described below.

Gels were transferred overnight at 4 °C or 2 h at RT onto Amersham Hybond electrochemiluminescence nitrocellulose membrane (GE Healthcare, Buckinghamshire, UK) and blocked for 15 min prior to 2 h incubation with primary antibody (1:5000 dilution, polyclonal rabbit anti-human tau A0024, Dako, Ely, UK). Unbound antibody was removed by 5 × 5 min washes with 10% TBS-Tween in distilled water before 2 h incubation at RT with secondary antibody (goat anti-rabbit IgG 31460, Thermo Scientific, UK, 1:5000 dilution) and the membrane washed as previously. For WB to confirm cleavage of the histidine tag, an anti-His antibody (27-4710-01 from GE Healthcare, 1:2500 dilution) and rabbit anti-mouse IgG (31450 from Thermofisher, 1:5000 dilution) were used as the primary and secondary antibodies respectively. Antibody detection was performed using the Amersham electrochemiluminescence detection reagents according to the manufacturer's instruction and bands visualised by exposure to X-ray film (Fuji Medical X-ray Film Super RX) and developed in an AGFA Curix 60 processor (Agfa Healthcare, Greenville, SC, USA). Densitometry analysis of protein bands was performed using ImageJ [18], and differences compared using the Mann-Whitney test in Prism 6 (GraphPad Inc., CA, USA) at 5% significance level.

### 2.6 Large-scale expression of tau constructs

Large cultures were produced by inoculating 500 ml selective LB broth with 10 ml overnight cultures using the expression parameters described above. Cultures were subsequently centrifuged at 4 °C for 10 min at 9800×*g*. The resulting pellet was resuspended in 50 mM Na_2_PO_4_ pH 7.5 and frozen until use. Prior to purification, the lysate was boiled at 70 °C for 10 min to thaw, and protease inhibitor cocktail (Roche Diagnostics, 1 tablet/∼50 ml lysate), DNAse I and 5 ml of 1× BugBuster^®^ protein extraction reagent (Merck Millipore) added. The mixture was left to stand at RT for 1 h, sonicated at 70% power for 1 min and centrifuged at 4 °C for 30 min at 48,000×*g*. The supernatant containing the soluble fraction (crude extract) was decanted, filtered through a 0.2 μm filter and purified as below.

### 2.7 Protein purification by immobilised metal affinity chromatography (IMAC)

A Ni-NTA column was used. Chelating sepharose resin (GE Healthcare, UK) was charged with 10 mM NiCl_2_/CH_3_COONa pH 4.0 and equilibrated with buffer A (50 mM Na_2_PO_4_ pH 7.0, 500 mM NaCl, 10 mM imidazole) before addition of the crude extract. The column was re-washed with buffer A, followed by buffer B (50 mM Na_2_PO_4_ pH 7.0, 500 mM NaCl, 25 mM imidazole) and the protein eluted with buffer C (50 mM Na_2_PO_4_ pH 7.0, 500 mM NaCl, 500 mM imidazole) followed by overnight dialysis in the presence of 25 μg/ml TEV protease, against dialysis buffer (50 mM Tris HCl pH 7.5, 100 mM NaCl) to cleave the polyhistidine-TEV tag. The dialysed tau was re-purified to isolate the TEV protease-cleaved tau. Briefly, the column was equilibrated with buffer A, the cleaved protein collected as the flow through upon elution and ultra-filtered using Vivaspin 20 (5 kDa cut off) where necessary. At each step of the purification process, aliquots were taken for SDS-PAGE and WB analysis. Protein concentration and purity were estimated using the Bicinchoninic acid assay (G-Biosciences, Missouri, USA) and SDS-PAGE respectively.

### 2.8 Preparation of Alzheimer-like PHF

A method adapted from Barghorn et al. (2005) [13] was used. Briefly, 200 μl reaction mixtures containing 12 μM tau, 10 mM Na_2_PO_4_ pH 7.4, 50 mM ammonium acetate, 1 mM DTT and 6 μM heparin (MW 6000) were prepared and incubated at 37 °C for ∼ 7 days to promote filament formation.

### 2.9 Circular dichroism (CD) spectroscopy

Following dialysis of the purified tau protein in Thermo Scientific™ Slide-A-Lyzer™ MINI Dialysis Device (10K MWCO) in excess of 10 mM Na_2_PO_4_ pH 7.4 buffer as per the manufacturer's instructions, 10 μM samples were diluted in the same buffer and CD spectra collected using a Jasco J-815 CD spectropolarimeter in a 1 mm path length cell. The response time was 1 s with a data pitch of 0.1 nm and the scan speed was 100 nm/min. 32 spectra accumulations were obtained for each sample at wavelengths ranging from 190 nm to 280 nm and averaged. The high tension voltage was ≤550 V throughout.

PHF samples were centrifuged at 100,000×*g* for 1 h at 4 °C, supernatant removed and the pellet resuspended in 200 μl of 10 mM Na_2_PO_4_ pH 7.4. CD analysis on PHF samples was conducted as described above. Secondary structure elements were estimated using the DichroWeb Contin-LL (Provencher and Glockner method) reference dataset 4 [19], [20], [21].

### 2.10 Transmission electron microscopy (TEM)

300-mesh Formvar carbon-coated copper grids (Agar scientific, UK) were glow-discharged and 5 μl of 1:2 dilutions of filament preparations pipetted onto the grid and allowed to bind for 1 min, excess sample removed and 5 μl uranyl acetate added for 1 min and excess removed. The grids were imaged using a JEOL-2100F transmission electron microscope.

## 3 Results

### 3.1 Preparation of tau pathogenic mutants

**Supplementary Fig. 1:**
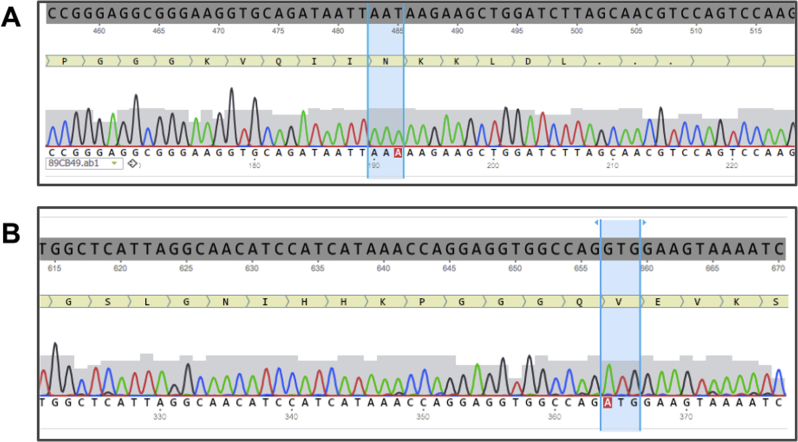
**Sequence alignments confirming the creation of two FTPD-17 mutations in the K18 WT tau construct. (A)** Sequence alignment showing the creation of the N279K point mutation (AAT (Asn) → AAA (Lys)). This pathogenic mutation is within the second tau repeat, present only in 4R isoforms. (**B)** Creation of the V337M mutation **(**GTG (Val) → ATG (Met)) in the third repeat region of tau. This mutation can affect both 3R and 4R tau isoforms due to its location in a constitutively-spliced region. Horizontal sequences shown in gray are those of the original construct (K18 WT). The corresponding amino acid sequence is shown in yellow underneath. Aligning nucleotide sequences from DNA sequencing are indicated under the chromatogram. Nucleotides highlighted in red indicate variation from the reference sequence. Sites of the mutations are highlighted in blue.

The K18 WT construct was used as a platform for the creation of tau constructs carrying the N279K and V337M FTPD-17 mutations. Both mutations were single point: N279K = AAT (Asn) → AAA (Lys) and V337M = GTG (Val) → ATG (Met), and were confirmed by DNA sequencing and subsequent multiple sequence alignments to the original K18 WT construct (Supplementary Fig. 1).

### 3.2 Comparison of tau expression in two different *E*. *coli* strains

**Fig. 2:**
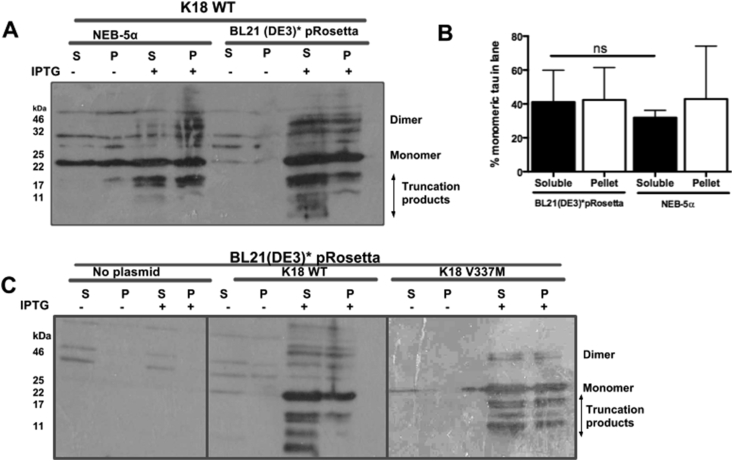
Representative Western blot analysis of K18 tau expression in the NEB-5α and BL21(DE3)*pRosetta *E coli* strains. (A) Dimerisation and truncation of expressed tau protein were noticeable in both strains. **(B)** Densitometry analysis showing that soluble monomeric tau levels in the BL21(DE3)*pRosetta strain and NEB-5α were not significantly different (two-tailed Mann Whitney test, p = 0.6). Data shown as mean ± standard deviation of three independent experiments. **(C)** Evaluation of K18 WT and K18 V337M expression in the BL21(DE3)*pRosetta strain. Comparable levels of tau expression, aggregation and truncation were observed in the soluble and pellet portions of both constructs, suggesting that the presence of the pathogenic mutation did not have detrimental effects on tau protein expression. S, soluble fraction; P, pellet (insoluble) fraction; IPTG, isopropyl β-D-1 thiogalactopyranoside.

Tau expression in two *E*. *coli* strains – BL21(DE3)*pRosetta and NEB-5α – was compared (Fig. 2). The intention was to compare expression in the former strain which is adapted for the expression of eukaryotic proteins, with the latter strain lacking this property. After IPTG induction, expressed tau proteins were positively identified with the anti-tau antibody used (Fig. 2A). SDS-resistant K18 WT monomers and dimers were approximately 22 kDa and 44 kDa in size respectively, with shorter fragments most likely indicating truncation products. The resistance of tau to denaturation by SDS has been previously reported [22], [23]. Note that the presence of the c-Myc, poly-histidine and TEV tags, and the anomalous SDS-binding property of tau influenced the observed size of purified tau. Higher amounts of soluble monomeric tau were recorded in the BL21(DE3)*pRosetta strain compared to NEB-5α, although this difference was not significant (two-tailed Mann Whitney test, p = 0.6; Fig. 2B). While comparable levels of monomeric tau were observed in the soluble and insoluble (pellet) fractions of BL21(DE3)*pRosetta (41.1 ± 18.8 and 42.4 ± 19.1 respectively), expression in NEB-5α resulted in higher levels of monomeric tau in the insoluble fraction compared to the soluble fraction (42.9 ± 22.1 and 31.9 ± 4.36 respectively; Fig. 2A and B), suggesting a higher proportion of inclusion body formation in this host. For this reason, all subsequent experiments were conducted using tau transformed into BL21(DE3)*pRosetta. Note that the densitometry analysis was focused on monomers as the most abundant and primary tau protein species but due to the high tendency of the protein to form aggregation and truncation products, this may not represent overall tau production in some cases.

The effect of the V337M disease mutation on protein expression was also measured, since this point mutation is sufficient to reduce the MT-binding ability of tau *in vitro* [9]. In this experiment, similar levels of monomeric tau expression were observed for both K18 WT and the V337M variant, suggesting that the pathogenic mutation did not affect expression levels (Fig. 2C). Furthermore, both proteins shared tendencies to form aggregates and truncation products, indicating that the spontaneous aggregation and truncation properties of tau were independent of this mutation (Fig. 2C).

### 3.3 Temperature optimisation for tau expression

Subsequently, conditions for protein expression in the BL21(DE3)*pRosetta strain were investigated. The following parameters were studied: post-induction expression temperature, length of induction, and media supplementation with glucose.

**Fig. 3:**
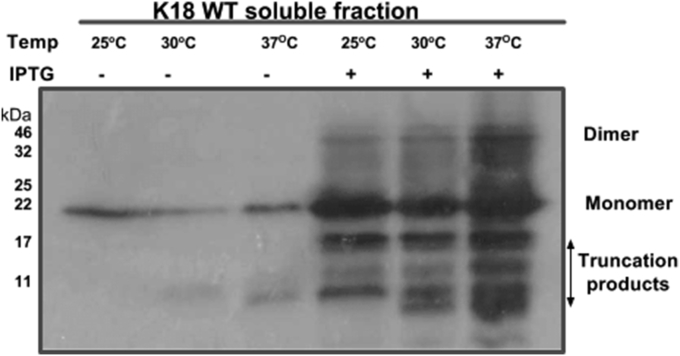
**Representative Western blot analysis showing the influence of induction temperature on expression levels of K18 WT tau**. Cultures of tau-encoding BL21(DE3)*pRosetta cells were grown at 37 °C until reaching OD_600_ = 0.6. After IPTG induction, the cultures were immediately split into three aliquots and grown at 25 °C, 30 °C or 37 °C for 3 h. The yield of soluble monomeric K18 WT was highest in cultures grown at 37 °C following induction, although aggregation and truncation also appeared favoured at this temperature.

To identify an ideal expression temperature for K18 production, an initial culture of K18 WT-expressing cells was divided into three equal aliquots immediately after induction and grown for a further 3 h at 25 °C, 30 °C and 37 °C. WB analysis showed that all three temperatures were suitable for tau expression. 37 °C was found to be the best for soluble tau production, as the expression of full-length monomeric K18 WT was highest at this temperature (Fig. 3). However, increasing temperature appeared to favour protein aggregation and truncation.

### 3.4 Induction length for optimum tau expression

**Fig. 4:**
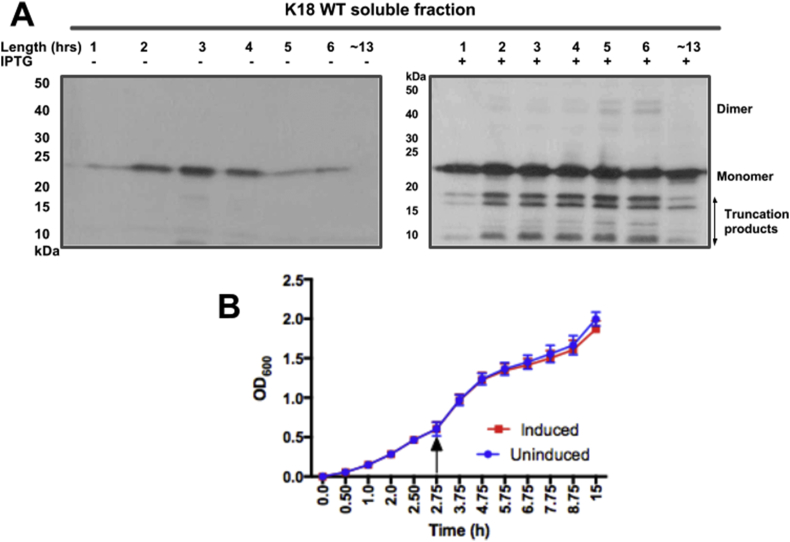
**Time-course evaluation of K18 WT expression in *E***. ***coli* using optical density measurement and Western blotting**. Cultures of tau K18 WT-expressing cells were grown at 37 °C until reaching OD_600_ = 0.6. The cultures were divided into two halves, and one portion induced with 0.5 mM IPTG. Both cultures were subsequently incubated at 37 °C, and OD_600_ readings and aliquots for Western blotting taken periodically. **(A)** Soluble cell fractions showing the expression of K18 WT over time. Truncation products were least evident at 1 h post-induction but increased after this time point. **(B)** Growth curves of IPTG-induced and uninduced cultures of tau, showing the same pattern of growth for both cultures. Arrow indicates point of induction.

Optimal incubation time can vary depending on the target protein and the choice of expression system. To establish an optimum induction length for K18 WT expression, aliquots of induced cultures were taken at regular intervals post-induction and analysed by WB. Although no marked difference in monomeric tau levels was recorded at the different timepoints, truncation products and aggregates were least evident at 1 h (Fig. 4A).

Next, the growth curves of uninduced and induced cultures were monitored to ascertain whether prolonged expression resulted in accumulating toxicity. Although the uninduced culture grew at a slightly faster rate post-induction, this difference appeared minimal, suggesting that tau expression did not pose significant cellular toxicity to the bacterial system (Fig. 4B).

### 3.5 Media supplementation with 0.2%w/v glucose

**Fig. 5:**
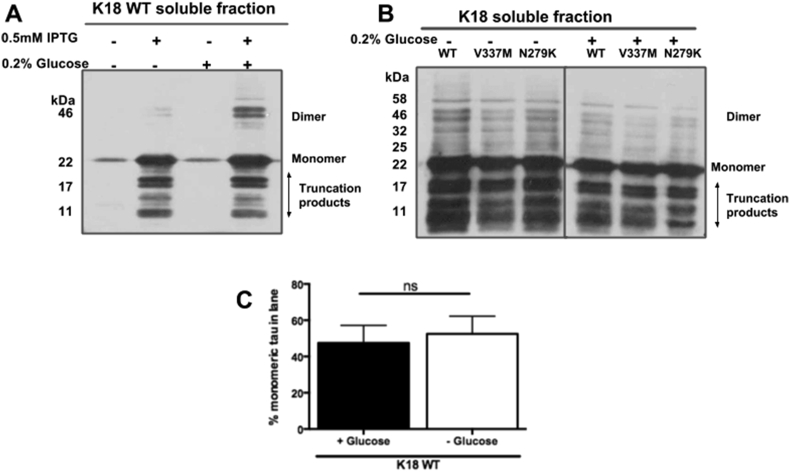
**Media supplementation with 0.2% glucose did not significantly enhance tau expression**. BL21(DE3)*pRosetta cells expressing tau were grown in selective LB media in the presence or absence of glucose. **(A)** Representative Western blot analysis of soluble cell fractions showing the expression of K18 WT in the absence and presence of 0.2% glucose. **(B)** Western blot analysis of induced protein expression in K18 WT and its FTPD-17 variants (V337M and N279K). **(C)** Densitometry analysis of monomeric K18 WT expression in LB media with or without glucose showed that there was no significant difference in protein expression (two-tailed Mann Whitney test, p = 0.6667, n = 3).

Glucose regulates the *lac* operon and may reduce promoter “leakiness” (detectable expression without induction) [24]. Glucose may therefore be provided as an extra carbon source to enhance growth of *E*. *coli* expressing a gene of interest. Addition of up to 1.0% glucose has been shown to enhance protein yield [25]. For this reason, the effect of media supplementation with 0.2% glucose prior to IPTG induction of tau expression was investigated. The level of monomeric K18 WT was not significantly enhanced by glucose supplementation (p = 0.6667; Fig. 5A and C). Similar findings were also recorded for K18 with the two FTPD-17 variants (Fig. 5B).

### 3.6 Purification of tau constructs

**Fig. 6:**
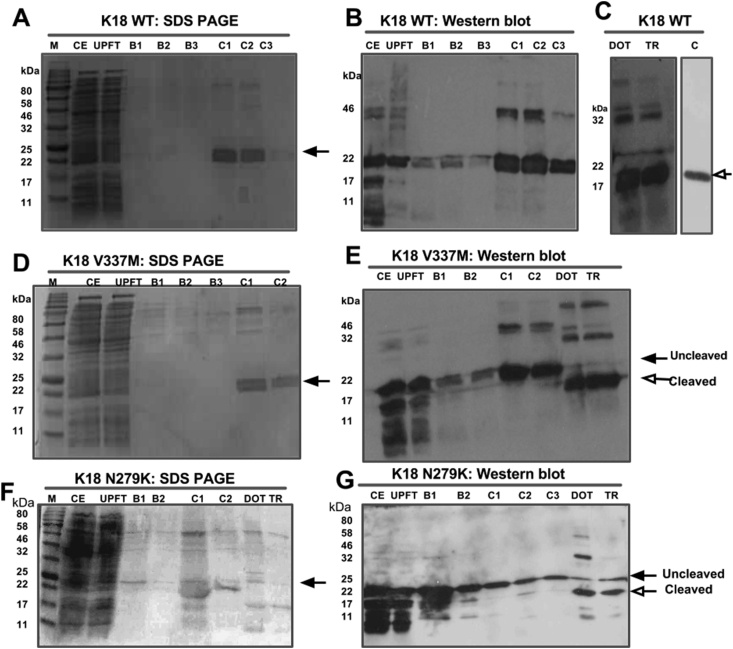
**Analysis of IMAC-based tau purification using SDS-PAGE followed by Western blotting**. Cultures were grown until reaching OD_600_ = 0.6, IPTG-induced and grown for a further 1 h after which the expressed tau protein was recovered and purified. Aliquots taken at each step of the purification process were analysed on 15% Tris-glycine SDS-PAGE gels. **(A), (D)** and **(F)** show SDS-PAGE analysis of the purification of K18 WT, K18 V337M and K18 N279K respectively. **(B)** and **(C)** are Western blots of K18 WT purification. **(E)** and **(G)** Western blot analysis of the purification of K18 V337M and K18 N279K respectively. Arrows indicate monomeric tau; black arrows point to uncleaved His-tagged tau while open arrows refer to tag-free tau. M = marker protein; CE = crude extract; UPFT = unbound protein flow-through; B1, B2 and B3 = wash fractions; C1, C2 and C3 = eluted fractions; DOT = pooled eluted fractions dialysed overnight in the presence of TEV protease, against dialysis buffer; TR = tau recovered from the second purification process after TEV cleavage. C = K18 WT control protein from a previous preparation after the removal of breakdown products and aggregates, leaving homogenous monomeric tau.

**Fig. 7:**
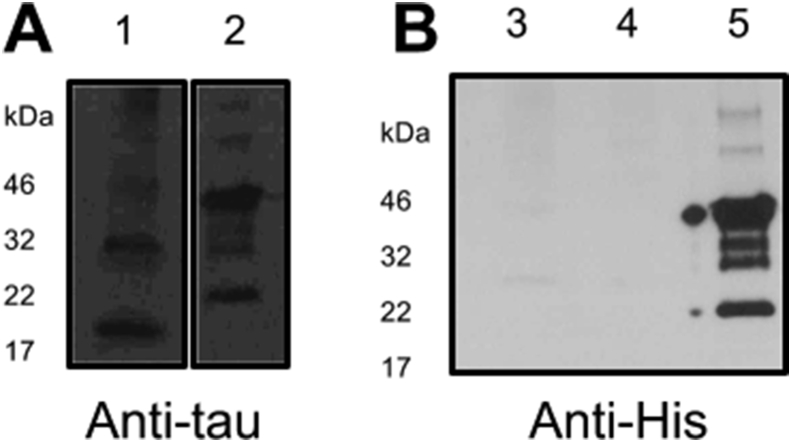
**Confirmation of cleavage of the polyhistidine tag. (A)** Western blotting using the total tau antibody (A0024 from Dako) confirmed the presence of tau in purified K18 WT with and without the His tag cleaved (lanes 1 and 2 respectively). **(B)** Western blotting using the anti-His antibody however recognised the His tagged K18 WT (lane 5) but not equal concentrations of K18 WT and K18 N279K with the His tags cleaved (lanes 3 and 4 respectively).

After identifying optimum conditions for expressing K18, these conditions were employed in growing larger cultures of tau that were subsequently purified using IMAC. All three protein constructs (K18 WT, K18 V337M and K18 N279K) were successfully purified using this system, followed by overnight dialysis and TEV site cleavage. Tau expression and purification were confirmed by SDS-PAGE and WB (Fig. 6). As expected, the crude extracts contained tau in addition to contaminating cellular proteins (lanes labelled CE in Fig. 6). After initial separation of the crude extracts, the tau component was bound to the Ni^2+^ resin, leaving unbound proteins to filter through (UPFT lanes in Fig. 6). Washing the column with buffer B removed residual contaminants and loosely-bound protein (lanes B1-B3 in Fig. 6). Proteins were eluted with buffer C containing 500 mM imidazole (C1-C3 lanes in Fig. 6). and subsequently dialysed overnight with TEV protease and the purification process repeated to isolate tau proteins with the TEV site cleaved. Complete cleavage of the His tag was confirmed in WB using an anti-His antibody (Fig. 7). While the total tau antibody recognised K18 WT with or without the His tag (Fig. 7A, lanes 2 and 1 respectively), the anti-His antibody had no recognition for K18 WT and K18 N279K with the His tags cleaved (Fig. 7B, lanes 3 and 4 respectively) but for K18 WT with the His tag intact (Fig. 7B, lanes 5). Protein yield was up to ∼5.5 mg/ml per 500 ml culture (Table 3). WB showed that the tau constructs were purified both in the monomeric and dimeric forms (and sometimes in higher oligomeric forms; Fig. 6), emphasising the characteristic tendency of the protein to readily form aggregates. Contrary to the expression experiments, truncation products were rarely observed in purified proteins.

### 3.7 CD assessment of secondary structure properties of the purified tau

**Fig. 8:**
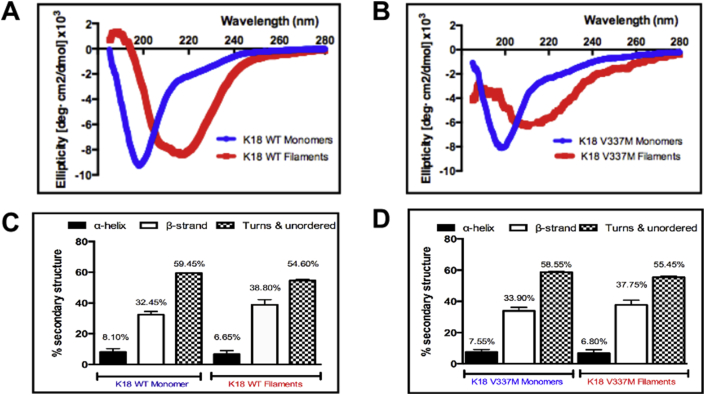
**Characterisation of the purified tau proteins using circular dichroism spectroscopy and the preparation of Alzheimer-like paired helical filaments. (A)** and **(B)** Circular dichroism spectra within the far-UV region were obtained from K18 WT and K18 V337M monomers (blue curves) and insoluble filaments (red curves). The spectra for monomers showed negative peaks at about 200 nm. Filament formation induced a positional shift in the peak towards 220 nm. **(C)** and **(D)** The circular dichroism data gathered were subjected to computational analysis to estimate secondary structure content. The CONTIN algorithm in the DichroWeb database predicted that the monomeric tau were predominantly random-coiled (turns and unordered). The preparation of paired helical filaments induced secondary structure re-organisation towards β-sheets. (For interpretation of the references to colour in this figure legend, the reader is referred to the web version of this article.)

Following successful purification of the tau proteins, K18 WT and K18 V337M were characterised to compare their biochemical properties to those previously reported [10], [26]. Firstly, CD spectroscopy was used to investigate secondary structure conformation of the purified tau. To assess the secondary structures of the tau proteins in solution, their CD spectra were measured in the far-ultraviolet spectral region (wavelength 190–280 nm) using proteins dissolved in 10 mM phosphate buffer (pH 7.4) at RT. Previous studies have reported that tau, in the mostly-monomeric state, is inherently unfolded, with predominantly random-coil conformation [4], [5], [10], [26], [27]. Our analysis confirmed this observation for the purified proteins; CD spectra for both K18 WT and K18 V337M showed minimum peaks around 200 nm (Fig. 8A and B), characteristic of predominantly random coil structures [10], [26]. Moreover, the depth of the negative peaks were similar for both the WT and mutant constructs, suggesting that the disease-associated mutation did not cause obvious changes in secondary structure of tau as previously reported [4]. To estimate the relative contribution of different secondary structure elements, the CD spectra were analysed with the CONTIN algorithm in DichroWeb, which showed that the secondary structures were dominated by random coils, with traces of α-helices and β-sheets (Fig. 8C and D).

### 3.8 Preparation of Alzheimer-like PHF and subsequent imaging using negative-stain TEM

**Fig. 9:**
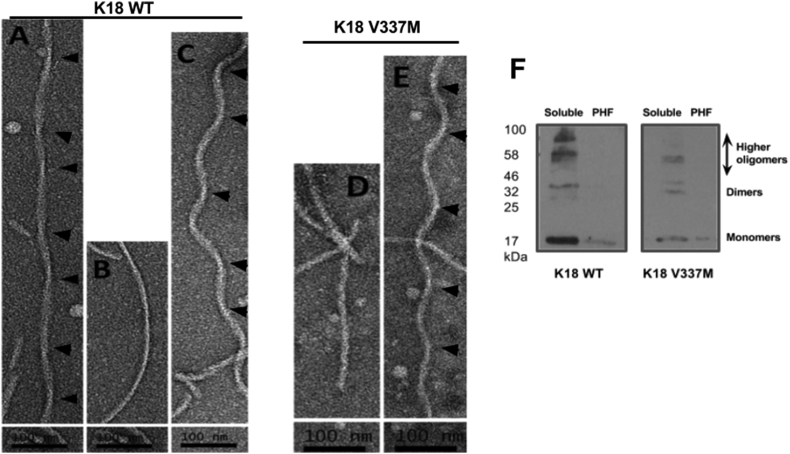
**Representative electron micrographs showing negative-stained Alzheimer-like filaments of distinct morphologies prepared from the purified tau proteins**. Panels **(A), (B) and (C)** are from K18 WT while (**D)** and (**E)** are from K18 V337M. Panel A shows paired helical filaments of varying width. Panels B and D contain straight filaments with no obvious periodicity. Panels C and E display spiral-like paired helical filaments. Arrowheads indicate possible peak-to-peak crossover points. **(F)** Following heparin-induced tau aggregation, filament samples were ultracentrifuged to isolate soluble species from insoluble aggregates (paired helical filaments; PHF). Western blot analysis suggested that the soluble fraction consisted of a heterogeneous mix of monomers and oligomers while the insoluble aggregates consisted mostly of higher molecular weight filaments that were not detectable by Western blotting.

In order to validate their authenticity, Alzheimer-like filaments were prepared from the recombinant proteins and visualised by TEM. PHFs were prepared in the presence of the reducing agent DTT and the polyanion inducer heparin. The reaction was carried out in 10 mM Na_2_PO_4_ pH 7.4 buffer. The use of a non-chlorine-containing buffer enabled subsequent CD investigations on the prepared PHF without having to change buffers as was previously done [10]. TEM-based structural examination suggested that the filaments consisted of distinct features including two strands wound round each other, with crossover points every few nanometers (Fig. 9) in line with previous observations [5], [28].

Subsequently, the filamented tau samples were analysed by CD to assess whether PHF assembly induced a shift in secondary structure composition as suggested [26]. The CD spectra for filamented tau showed a shift in peak to approximately 220 nm, which is indicative of β-sheet formation (Fig. 8). While the soluble fractions in the PHF preparation samples consisted of monomers and low molecular weight oligomers, the insoluble PHF was predominantly made up of higher molecular weight species that were not noticeable by WB (Fig. 9F), consistent with previous observations [29].

## 4 Discussion

Tau protein has been the subject of intensive research because of its physiological importance in stabilising MTs, supporting axonal transport, neurite outgrowth and other processes involved in neuronal cell biology [2], [30], [31]. In addition, toxic aggregation of WT tau and its FTPD-17 variants is the primary hallmark of Alzheimer's disease and other tauopathies [2]. Efficient production of recombinant tau is a pre-requisite for the *in vitro* and some *in vivo* modelling of these diseases. Insufficient yield of this target protein can result in several experimental pitfalls. In this study, we investigated parameters that can affect the expression and purification of WT and FTPD-17 pathogenic tau, in an attempt to identify ways to increase the yield of pure tau for downstream applications. It was shown that the presence of the V337M and N279K pathogenic mutations appeared not to affect monomeric tau expression, which was greatest at 37 °C induction temperature compared to 25 °C and 30 °C. Build-up of truncation products and aggregates was least evident at 1 h post-induction, compared to up to 13 h post-induction. Supplementing the growth medium with 0.2% glucose did not significantly affect the percentage of monomeric tau present. The expressed protein was purified using IMAC and characterised by CD and PHF analysis. Our findings show that the described method led to the production of authentic tau proteins, which followed the known biochemical and biophysical properties. This report provides a reliable method for efficient expression and purification of tau that would be useful for further studies.

The BL21(DE3)* pRosetta strain used in this study was deficient in RNAseE, and genetically modified to be Lon and OmpT protease-deficient, making it suitable for high-yield expression of foreign genes in bacteria. WB showed that using this strain did not significantly enhance the amount of monomeric tau compared to NEB-5α (Fig. 2). This was possibly due to the high propensity of tau to aggregate and form truncated peptides, thus preventing efficient evaluation of monomeric tau yield. Whilst 37 °C appeared optimal for expression of monomeric tau, aggregation seemed to increase with temperature (Fig. 3). This corroborates previous reports that tau polymerisation is most efficient at high temperatures [14]. A relatively short induction time of 1 h was optimal for the amount of monomeric protein yields achieved and a concentration of 0.1 mM – 1 mM IPTG was sufficient to induce expression (data not shown). Media supplementation with glucose did not significantly enhance expression yield, suggesting that the strain can be used for tau production without glucose (Fig. 5). A common observation throughout the expression-optimisation experiments was the high tendency of the tau constructs to form truncation products, in both strains studied (Fig. 2, Fig. 3, Fig. 4, Fig. 5). This possibly could have been reduced by the addition of protease inhibitors to the growth medium or lysis buffer, notwithstanding the reported protease resistance of the tau repeat domain [32]. However, this step was omitted to allow the proteins to be observed in a native-like state. It was also observed that a considerable portion of the protein existed in the pellet fraction, which could not be recovered by additional sonication (Fig. 2). This was likely as a result of the formation of inclusion bodies or insoluble aggregates and not due to insufficient cell lysis, as reported for another protein [33].

The purified tau proteins were characterised using CD spectroscopy, PHF preparation and TEM visualisation of PHFs (Fig. 7). The CD data revealed that the purified tau proteins were inherently unfolded, showing negative peaks at approximately 200 nm. For direct evidence of filament formation, samples were negative-stained and imaged by TEM, which indicated that the purified tau proteins were capable of forming filaments. Filament formation was induced with the addition of heparin; the role of heparin as an inducer of PHF formation has been characterised [34]. Filament formation is promoted by hexapeptide motifs at the beginning of the second and third tau repeats [35], both of which were intact in the 4R tau constructs used [27]. Fig. 8, Fig. 9 indicate that the tau proteins produced were able to form filaments as expected, and that the filaments consisted of high molecular weight oligomers compared to pre-filament species. The observed structural shift in CD spectra following tau aggregation can also be achieved by incubating tau at physiological temperatures with or without heparin, although incubation times are lengthened in the absence of heparin [27], [36].

An advantage of the method reported here is that it enables the production of tau oligomers and monomers (the toxicity of both of which is currently under intense investigation) through one- or two-step purification protocols. The first purification step produces polyhistidine-tagged tau; the tag can be removed following TEV protease treatment during dialysis. We have confirmed that both His-tagged and tag-free tau proteins prepared using the described protocol are fully functional in terms of their aggregation tendency, ability to form Alzheimer-like filaments, and secondary structure features. These findings are in agreement with earlier studies that reported the use of untagged and His-tagged tau for *in vitro* and cell culture investigations [37], [36], [38], [39], further confirming the authenticity of the proteins produced using our simplified protocol.

Whether or not the purified tau should undergo further processing before utilisation for functional studies would depend on the intended use. The preparation of monomer-oligomer mixtures, described in this report, could be used for many studies. However, if exclusively monomeric tau is required (as in Fig. 6C lane C), then it is possible to remove truncation products and aggregates by size exclusion chromatography. It is important to be certain about the nature of purified tau being used for downstream applications (whether consisting exclusively of monomers, oligomers or a heterogenous mixture of the two) since seemingly conflicting results have been reported for the toxicity-seeding potential of monomeric and oligomeric tau [7], [8], [40].

Historically, there has been a debate as to which tau protein species is the most toxic to cells – whether monomers, oligomers, PHF or NFT [41], [42]. Recent attempts to address this question have also sought to understand if tau toxicity and pathology spread in a prion-like manner. In such studies, recombinant forms of the various protein species are injected into the brains of living experimental animals or extracellularly added to neuronal or neuron-like cells, and their ability to be internalised, secreted and co-aggregate with endogenous proteins monitored in real time [43]. Converging evidence has mostly suggested that oligomeric tau are the early-stage species required to initiate the trans-cellular spread, toxicity and associated learning and memory impairments [8], [44], [45], although others have reported that tau monomers are the fundamental toxic species [7]. Although the transcellular spread of tau pathology has become a well-known, experimentally-confirmed phenomenon, the mechanisms through which this process occur have however remained elusive. It was recently reported that the internalisation of tau K18 fibrils occurs via binding with heparan sulphate proteoglycans [46] whilst the extracellular release of 4R0N tau occurs by exocytosis mediated by interaction with the DnaJC/Hsc70 molecular chaperone complex [47]. It however remains unknown as to whether all tau isoforms and fragments and their frontotemporal dementia variants are internalised and secreted through these same processes. As the scope of research in this area grows, the importance of recombinant tau will undoubtedly increase. Certainly, recombinant tau proteins of the highest purity and yield will be essential to produce reliable and reproducible data in future studies investigating the cellular mechanisms involved in the spread of tau toxicity as well as the *in vitro* structural, biochemical and biophysical aspects of the protein's ability to cause disease. The present study has demonstrated a simple protocol for the preparation of tau K18 proteins for such investigations. This is the first report that focuses specifically on the expression and purification characteristics of K18 tau. Although Barghorn et al. [13], earlier described a method that combines gel permeation and size exclusion chromatography for the purification of tau isoforms and constructs, a general protein yield range was provided (10–100 mg/10 L culture), with no specific value given for K18 tau. Protein yields for the tau constructs and isoforms vary [13], possibly due to the different structural and functional characteristics exhibited by these proteins. For this reason, studies dedicated to documenting expression and purification approaches for each tau protein is necessary to inform future research. Such reports are available for the adult human brain tau isoforms and some truncation constructs (Table 3), although the procedures described mostly depend on methodologies that are unavailable in many labs. In contrast, the protocol described in the present study makes use of a simple, inexpensive approach to purify both WT and mutant K18 tau proteins in high yield, without compromising on the purity. This protocol can be adapted for the preparation of the other tau proteins. In fact, we have successfully used this protocol in our laboratory to express and purify several other tau proteins, including K18 and the full length isoform carrying FTPD-17 mutations not described here, with similar protein quality and yield (data not shown).

## 5 Conclusion

We have investigated specific parameters affecting the production of the recombinant tau protein, reporting on conditions that may ensure optimal expression. Using these parameters, the tau MT repeat domain (K18) and two frontotemporal dementia variants were expressed and purified, leading to pure proteins of high concentrations. The purified proteins were natively unfolded and followed the classical fibrillisation pathway to form mature PHFs. The formation of filaments initiated a shift in secondary structure re-organisation from mostly random coils towards β-sheet formation. As such, this report demonstrates efficient methods for the production of tau protein that can be used in *in vitro* and *in vivo* studies.

## Competing interests

None.

## Author contributions

TKK, DAN, EJH and KGM designed the research; TKK, DAN, AT, RS, LCYL and BW conducted the research and analysed data; TKK, DAN, EJH and KGM wrote the paper.

## Figures and Tables

**Table 1:** Primers used in site-directed mutagenesis of the wild type K18 construct to create the FTPD-17 pathogenic mutations N279K and V337M. Mutated codons are underlined.

Primer	Sequence (5′ to 3′)
N279K_Forward	GCAGATAATTAAAAAGAAGCTGGATCTT
N279K_Reverse	ACCTTCCCGCCTCCCGGC
V337M_Forward	AGGTGGCCAGATGGAAGTAAAATC
V337M_Reverse	CCTGGTTTATGATGGATGTTG

**Table 2:** DNA sequencing primers.

Primer	Sequence (5′ to 3′)
Sequencing primer 1	ACAGACCATGTCGTACTACC
Sequencing primer 2	AGGCGGCAGTGTGCAAATAG

**Table 3:** Summary of studies on expression and purification of recombinant tau.

Study	Tau isoform or construct studied	Purification method used	Yield	Purity
This work	Wild type K18 and its N279K and V337M frontotemporal dementia variants	Ni^2+^ affinity chromatography	Up to ∼5.5 mg/ml, 4.0 mg/ml and 3.5 mg/ml protein per 500 ml culture for K18 WT, K18 V337M and K18 N279K respectively	Up to 99% purity for monomer only and 95% for monomer-oligomer mix
Tepper et al. (2014) [39]	2N4R (longest tau isoform in the adult human brain)	Tau expressed in Sf9 cells was purified by size exclusion and anion exchange chromatography	5–10 μM (subsequently concentrated to ∼50 μM)	Not indicated
Geodert and Jakes (1990) [23]	The six human tau isoforms	Ammonium sulphate precipitation and ion exchange chromatography	30–60 μg/ml culture	Up to 95% purity
Barghorn et al. (2005) [13]	Tau isoforms and constructs including K18	Gel permeation and cation exchange chromatography	10–100 mg protein/10 L culture (specific concentration for K18 not indicated)	95% for monomer-oligomer mixtures and 99% for monomer only
Csokova et al. (2004) [17]	221-441 fragment of tau	Cation exchange, anion exchange and size exclusion chromatography	4–8 mg protein/500 ml culture	Up to 98% purity

## Abbreviations

APS: ammonium persulphate
CD: circular dichroism
FTPD-17: frontotemporal dementia with Parkinsonism linked to chromosome 17
IMAC: immobilised metal affinity chromatography
IPTG: isopropyl β-D-1 thiogalactopyranoside
LB: Luria-Bertani
MT: microtubule
NFT: neurofibrillary tangle
PHF: paired helical filaments
PBS: phosphate buffered saline
SDM: site-directed mutagenesis
SDS: sodium dodecyl sulphate polyacrylamide gel electrophoresis
TEM: transmission electron microscopy
TEMED: tetramethylethylenediamine
TEV: Tobacco Etch Virus
WB: Western blotting
WT: wild type
